# PP94 Chronic Obstructive Respiratory Disease Patients’ Involvement In The Evaluation Of Transcutaneous Capnography In Primary Care

**DOI:** 10.1017/S0266462324002599

**Published:** 2025-01-07

**Authors:** Begoña Rodríguez-Ortiz-de-Salazar, Mario Cardaba-Arranz, Matilde Palma-Ruiz, D Nicole Hass, Luis-Maria Sanchez-Gomez, Jesús González-Enríquez

## Abstract

**Introduction:**

Patient participation in health technology assessment (HTA) plays an increasingly relevant role due to increased recognition of its essential contribution to addressing uncertainties in evidence and its real-world application. The objective is to analyze and describe how patients with chronic obstructive pulmonary disease (COPD) participate in the evaluation of transcutaneous capnography (TC) in the primary care setting.

**Methods:**

The Spanish Association of Patients with Chronic Obstructive Pulmonary Disease facilitated contact with three COPD expert patients. A face-to-face video interview was conducted with each patient to know about their knowledge of the technology, real-life experiences, and expectations. Patients were informed of the objective of the evaluation and signed confidentiality and conflict of interest forms. All interviews were conducted in April 2023 by two researchers. Expert patients with COPD were able to participate in the review of the protocol and in the final version of the report. Literature searches were also conducted on patient perceptions of TC compared to arterial blood CO_2_ measurement.

**Results:**

All patients were male, older than 60 years and were ex-smokers with greater than 30 years of tobacco consumption. Patients highlighted the relevance of early detection of COPD to facilitate the planning and organization of treatment as their clinical situation progresses. Likewise, they also emphasized the importance of the implementation of less invasive tests, and the proximity and accessibility of primary care. Two studies reported greater patient satisfaction and less pain with TC than with arterial blood gases.

**Conclusions:**

Expert patients show high expectations for the technology, as it is less invasive than arterial blood gases; they also acknowledged its proximity in primary care settings and the potential for detecting complications of oxygen therapy. Patient participation in HTA adds intangible value, as they provide “disease-specific knowledge” and real-life applicability of the technology.

